# Multisystem Inflammatory Syndrome in Children Temporally Related to COVID-19: A Case Report From Saudi Arabia

**DOI:** 10.7759/cureus.10589

**Published:** 2020-09-22

**Authors:** Heba H Al Ameer, Sajjad M AlKadhem, Fadi Busaleh, Sami AlKhwaitm, Maria Blesilda B Llaguno

**Affiliations:** 1 Infectious Diseases, Maternity and Children Hospital, Al-Ahsa, SAU; 2 Pediatrics, Maternity and Children Hospital, Al-Ahsa, SAU; 3 Pediatric Critical Care Medicine, Maternity and Children Hospital, Al-Ahsa, SAU; 4 Nursing, College of Applied Medical Sciences, King Faisal University, Al-Ahsa, SAU

**Keywords:** multisystem inflammatory syndrome, covid 19, kawasaki disease, kawasaki disease shock syndrome

## Abstract

The World Health Organization is still revising the epidemiology of multi-system inflammatory syndrome in children (MIS-C) and the preliminary case definition, although there is a dearth of robust evidence regarding the clinical presentations, severity, and outcomes. Researchers, epidemiologists, and clinicians are struggling to characterize and describe the disease phenomenon while taking care of the diseased persons at the forefronts. This report tackles the first case of a 13-year-old Saudi female with the MIS-C mimicking Kawasaki disease. Her main manifestations were fever, gastrointestinal symptoms, evidence of organ failure with an increase in inflammatory markers, and a history of coronavirus disease (COVID-19) infection. She had glucose-6-phosphate dehydrogenase (G6PD) deficiency and no significant previous history of any disease. She presented with signs of acute illness: high-grade fever (39.6°C) for five days accompanied by sore throat, malaise, reduced oral intake, abdominal pain, diarrhea, skin rash, bilateral non-suppurative conjunctivitis, and erythematous, cracked lips. Eventually, she died despite aggressive management based on the Centers for Disease Control and Prevention and the Saudi Ministry of Health guidelines for COVID-19 management. Based on this case, we suggest that pediatricians need to be aware of such atypical presentations and early referral to tertiary care is imperative for further early diagnosis and management.

MIS-C is a rare yet severe and highly critical complication of COVID-19 infection in pediatrics, leading to serious and life-threatening illnesses. Knowledge about the wide spectrum of presenting signs and symptoms and disease severity, including early detection and treatment, is pivotal to prevent a tragic outcome.

## Introduction

Citizens of Wuhan, China, were exposed to initial cases of pneumonia of unknown origin in December 2019 [1]. The causative organism of this pneumonia was identified as severe acute respiratory syndrome coronavirus 2 (SARS- CoV-2), a novel β-RNA coronavirus, named by the World Health Organization (WHO) as coronavirus disease 2019 (COVID-19) [2]. In April 2020, the United Kingdom National Health Service observed a nationwide increase in the multi-system inflammatory syndrome, probably linked with SARS-CoV-2. Riphagen et al. reported the laboratory and clinical features of a group of eight children who suffered hyperinflammatory shock and were also SARS-CoV-2 positive [3]. Clinical features of these cases were relatable with the characteristics of Kawasaki disease (KD), toxic shock syndrome (TSS), and the KD shock syndrome. There were also recent reports about clusters of children and adolescents with the same manifestations who required admission to intensive care units in the New York City Department of Health [4], Europe, and North America [5]. The number of critical cases who developed hypoxia and pediatric acute respiratory distress syndrome and required invasive mechanical ventilation represents 0.6% in a large retrospective study of Dong et al. [6]. Nonetheless, there is a possible relation between the pro-inflammatory syndrome with features of KD or TSS in children and the COVID-19. Recently, the Centers for Disease Control and Prevention defined this hyperinflammatory syndrome and termed it as a multi-system inflammatory syndrome in children (MIS-C) [7]. Data show that COVID-19 is uncommon in children; only 2% of cases are patients younger than 20 years of age [8]. This is a report of the first case of MIS-C related to COVID-19 disease in Saudi Arabia.

## Case presentation

**Figure 1:**
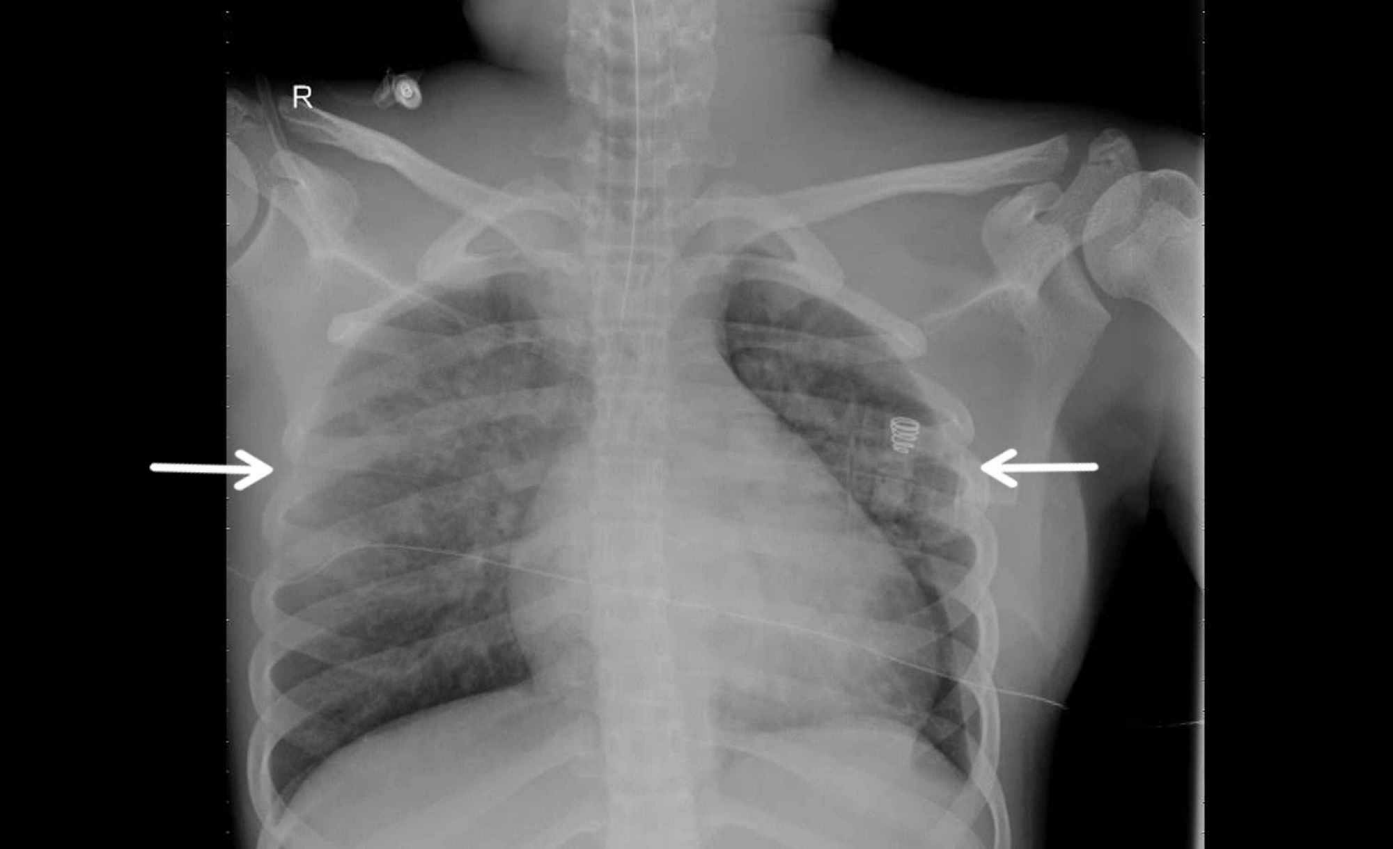
The patient’s chest X-ray is showing acute whiteout of the lung bilaterally consistent with acute respiratory distress syndrome. The X-ray is also showing that the patient is intubated.

Our case is a 13-year-old Saudi female with G6PD deficiency and no other significant medical history who presented to the emergency department with a five-day history of high-grade fever (39.8°C) accompanied by sore throat, malaise, abdominal pain, diarrhea, and reduced oral intake. Initially, the patient was conscious, alert, and vitally stable. Her physical examination revealed skin rash, bilateral non-suppurative conjunctivitis, and erythematous, cracked lips. The previous history revealed that the patient had a positive result from the nasopharyngeal reverse transcription-polymerase chain reaction (RT-PCR) for SARS-CoV-2 during a contact-tracing procedure. She was contacted by her mother who works as a health care provider in one of the quarantine facilities. She showed no symptoms during the quarantine period and hence was considered cured based on the MOH protocol (completing 10 days without symptoms from the first positive RT-PCR). She remained asymptomatic until day 23 when she developed fever, sore throat, abdominal pain, vomiting, and diarrhea; no consultation was done until two days after, where she was seen in the outpatient department and again tested positive for nasopharyngeal RT-PCR for SARS-CoV-2. She was given antibiotics and antiemetic drug and then was sent home. The persistence of symptoms for another two days prompted them to go to the emergency department, where she was subsequently admitted. Table 1 summarizes the investigations conducted upon admission. On the second day of admission, her condition deteriorated, manifesting tachypnea and tachycardia (heart rate of 130 beats per minute) and hypotension (blood pressure [BP] of 66/32 mmHg), with delayed capillary refill time (4 seconds) and decreased level of consciousness, suggestive of cardiogenic shock. The patient was given a normal saline bolus of 60 mL/kg /hour and was transferred to the pediatric critical care unit (PICU) as a case of pediatric multi-system inflammatory syndrome temporally associated with SARS-CoV-2 mimicking KD (Kawa-COVID-19). The abdominal ultrasound revealed mesenteric lymphadenitis; chest X-ray showed borderline cardiomegaly with clear lung fields, but a follow-up X-ray showed interval changes, as shown in Figure 1). Echocardiogram revealed mild mitral regurgitation, mild pericardial effusion, and moderate depression in left ventricle function (ejection fraction: 32%). Cardiac enzymes (high-sensitivity troponin) were elevated (0.454 ng/mL), suggesting myocarditis. Inotropic support with adrenaline and milrinone was started, and respiratory support with a high-flow nasal cannula (HFNC) with 2 L/minute flow was initiated. Overall clinical features (fever for five days, skin rash, erythematous cracked lips, bilateral non-suppurative conjunctivitis, extremity edema, elevated ESR (erythrocyte sedimentation rate), hypoalbuminemia, myocarditis, and mesenteric lymphadenitis) suggested atypical KD. IV immunoglobulins 2 g/kg single dose and methylprednisolone 2 mg/kg/dose twice daily were administered since the first day of admission. She was intubated and mechanically ventilated on her second day of PICU stay due to a new-onset bilateral patchy lung infiltrates with mild-to-moderate bilateral effusion associated with worsening hypoxia. She was managed according to the COVID-19 management protocol in Saudi Arabia, including antiviral favipiravir, tocilizumab interleukin-6 (IL-6) inhibitor, and low molecular weight heparin. However, by the third day, she was already on high-dose milrinone, epinephrine, norepinephrine (with unstable BP), on high ventilator settings, with persistent mixed acidosis, and started showing evidence of acute kidney injury as manifested by a BUN (blood urea nitrogen) level of 22 mmol/L, serum creatinine level of 500 mmol/L, and oliguria. Continuous renal replacement therapy (CRRT) was then initiated, but, subsequently, hepatic failure with coagulopathy ensued over the following days. On her sixth day in PICU, she suffered refractory hypotension primarily because of myocardial dysfunction until she had her terminal cardiac arrest.

**Table 1:** Blood investigations upon admission CBC, complete blood countLDH, Lactate dehydrogenase; AST, aspartate aminotransferase; ALT, alanine aminotransferase; PT, prothrombin time; PTT, partial thromboplastin time; INR, international normalized ratio; ESR, erythrocyte sedimentation rate; CRP, C-reactive protein; COVID-19, coronavirus disease 2019; IgG, immunoglobulin G; CMV, cytomegalovirus; EBV, Epstein-Barr virus; HSV, herpes simplex virus

Investigations	Reference Range	Patient Result
CBC		
Total white blood cells	5-15 ×10^3^/µL	15
Hemoglobin	10.5-14.0 g/dL	10
Platelet	150-400 × 10^3^/µL	200
Biochemistry		
Random serum glucose	74-160 mg/dL	127
Creatinine	53-115 µmol/L	102
Urea	1.7-8.30 mmol/L	5
Ca+	2.1-2.6 mmol/L	1.71
Na+	135-145 mmol/L	125
K+	3.5-5 mmol/L	3.0
CL-	95-115 mmol/L	103
Mg+	0.60-1.2 mmol/L	0.70
SO_4_+	0.81-1.85 mmol/L	0.63
LDH	100-190 units/L	258
Total serum bilirubin	0.2-1.0 mg/dL	0.754
Direct bilirubin	0.0-0.2 mg/dL	0.26
Albumin	34.0-50.0 g/L	28.7
AST	15-37 units/L	44
ALT	14-59 units/L	44
Alkaline phosphatase	46-116 units/L	110
Blood gases		
pH	7.35-7.45	7.38
HCO_3_	21-28 mmol/L	21
PCO_2_	35-45 mmHg/L	34
Coagulation profile		
PT	11.0-13.5 seconds	22
PTT	30-40 seconds	38
INR	0.8-1.1	1.40
Inflammatory markers		
ESR	0.0-29 mm/hour	101
CRP	Not available	
Ferritin	12-150 ng/mL	800
Cultures, serology		
COVID-19 IgG	Negative	Positive (70 mg/dL)
CMV-EBV, HSV	Negative	Negative
Blood culture, urine culture	Negative	Negative

## Discussion

**Figure 2:**
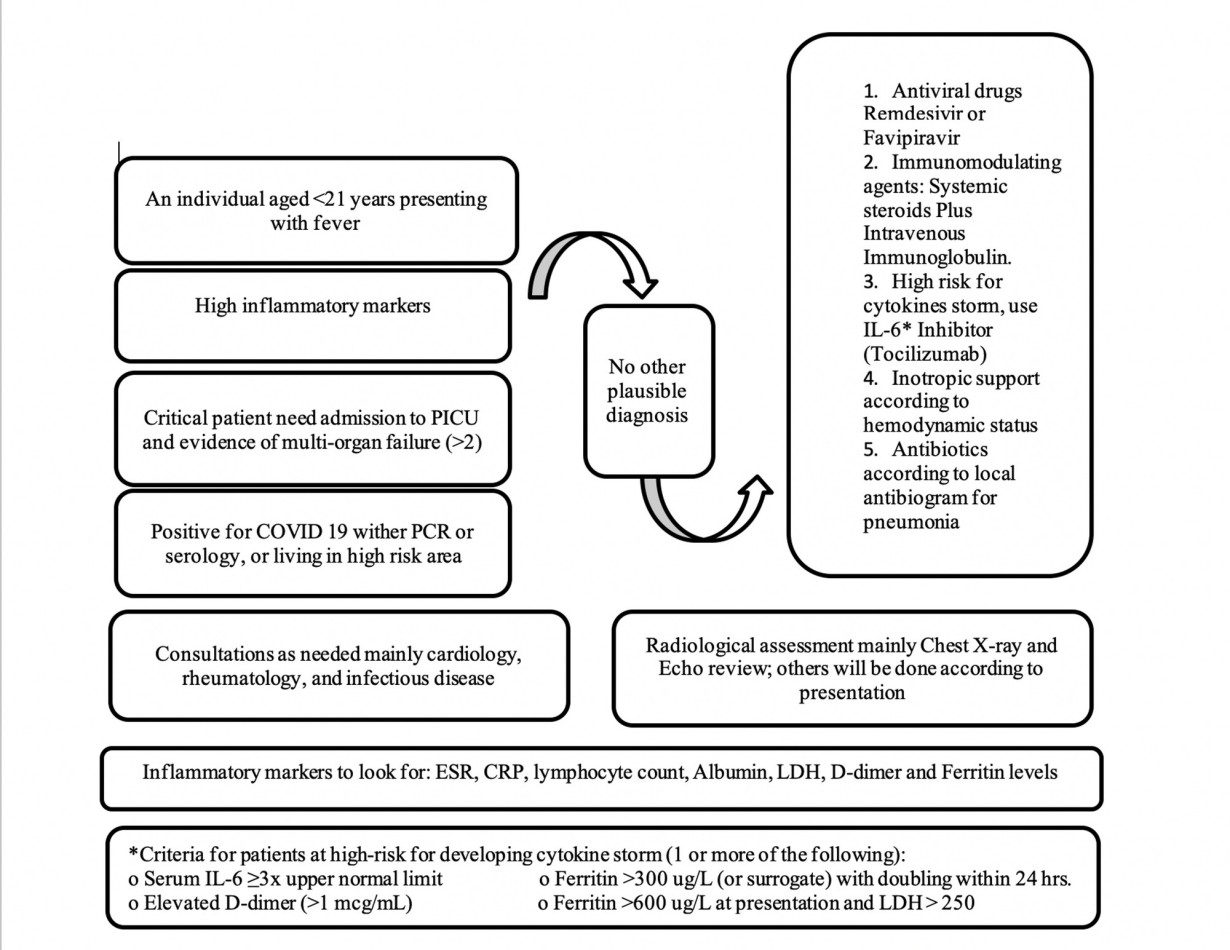
Brief steps from diagnosis until management of MIS-C due to COVID-19 from both the CDC and the Saudi Ministry of Health Guidelines. MIS-C, multi-system inflammatory syndrome in children; COVID-19, coronavirus disease 2019; CDC, Centers for Disease Control and Prevention

The term “hyperinflammatory shock in children” was first used by Riphagen et al. and was represented by clinical and laboratory features associated with COVID-19 virus infection. These cases usually progressed to shock and require intensive care unit admission [3]. Since there is a progressive increase in such cases globally, it led to the emergence of multiple similar definitions by different global organizations. The main manifestations were fever, gastrointestinal symptoms, and evidence of organ failure with an increase in inflammatory markers and evidence of COVID-19 infection or recent contact with the COVID-19 patient, with no other explanation of such presentation [3-6]. All of such were observed in our patient as she came with fever, diarrhea, vomiting, and abdominal pain, which progressed to circulatory failure and ended up by multiple organ failure. It is entirely possible that coronavirus infection in the recent past can trigger an immune response simulating KD, but the former has a later age of presentation and presents with severe cardiac involvement and cardiogenic shock, with almost no coronary arteries involvement [8], which were all manifested by our patient. The patient was managed to utilize the COVID-19 management protocol by the Saudi Ministry of Health Protocol for Patients Suspected of/Confirmed with COVID-19 [9], starting with favipiravir as an antiviral drug to cover the viral replication in both the respiratory and non-respiratory tissues and immunomodulatory agents, which include immunoglobulin and systemic corticosteroids for immune-mediated injury. In addition, supportive care was also carried out, such as vasoactive drugs, advanced mechanical ventilation, parental nutrition, and dialysis by CRRT. Tocilizumab, an IL-6 inhibitor, which is usually used for cytokines storm, was administered to the patient since she manifested persistent fever and high inflammatory markers (erythrocyte sedimentation rate, ferritin, and lactate dehydrogenase) [10-12]. However, the IL-6 level could not be measured because it was not available. Unfortunately, the patient deteriorated in a short time. This is the first report from Saudi Arabia on MIS-C associated with COVID-19 infection. Although there is still a dearth of robust evidence regarding its clinical presentations, severity, outcomes, and epidemiology, the WHO has already developed a preliminary case definition for this condition, which will be revised as more data become available. Still, pediatricians need to be aware of such atypical presentations, and early referral to tertiary care is imperative for further early diagnosis and management (Figure 2).

## Conclusions

MIS-C is a rare yet severe and highly critical complication of COVID-19 infection in pediatrics, leading to serious and life-threatening illnesses. Knowledge about the wide spectrum of presenting signs and symptoms and disease severity, including early detection and treatment, is pivotal to prevent a tragic outcome.
